# A Survey of Vision-Based Human Action Evaluation Methods

**DOI:** 10.3390/s19194129

**Published:** 2019-09-24

**Authors:** Qing Lei, Ji-Xiang Du, Hong-Bo Zhang, Shuang Ye, Duan-Sheng Chen

**Affiliations:** 1 Department of Computer Science and Technology, Huaqiao University, Xiamen 361000, China; zhanghongbo@hqu.edu.cn (H.-B.Z.); shuangy_amoy@163.com (S.Y.); dschen@hqu.edu.cn (D.-S.C.); 2 Xiamen Key Laboratory of Computer Vision and Pattern Recognition, Huaqiao University, Xiamen 361000, China

**Keywords:** human action evaluation, action quality assessment, feature learning, hand-crafted features, deep learning features, action evaluation dataset

## Abstract

The fields of human activity analysis have recently begun to diversify. Many researchers have taken much interest in developing action recognition or action prediction methods. The research on human action evaluation differs by aiming to design computation models and evaluation approaches for automatically assessing the quality of human actions. This line of study has become popular because of its explosively emerging real-world applications, such as physical rehabilitation, assistive living for elderly people, skill training on self-learning platforms, and sports activity scoring. This paper presents a comprehensive survey of approaches and techniques in action evaluation research, including motion detection and preprocessing using skeleton data, handcrafted feature representation methods, and deep learning-based feature representation methods. The benchmark datasets from this research field and some evaluation criteria employed to validate the algorithms’ performance are introduced. Finally, the authors present several promising future directions for further studies.

## 1. Introduction

**Figure 1:**
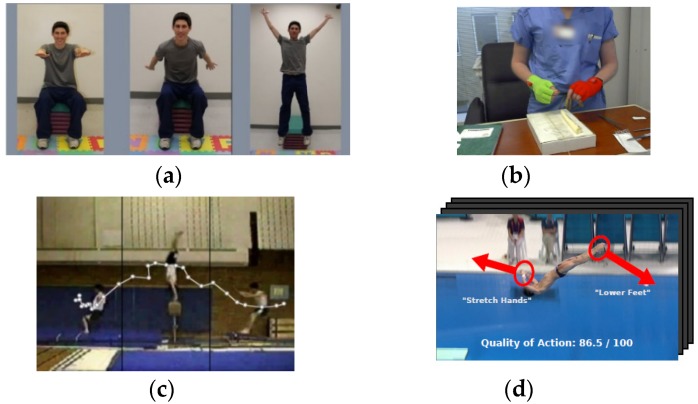
Several application fields of human action evaluation. (**a**) healthcare and rehabilitation; (**b**) surgical skill training; (**c**) sports skill training; (**d**) sports activity scoring.

In recent years, human action evaluation has emerged as an important problem in a variety of computer vision applications, which range from sports training [1,2,3,4,5] to healthcare and physical rehabilitation [6,7,8,9], interactive entertainment [10,11,12], and video understanding [13,14,15]. In contrast to the aims of traditional human action recognition to infer the class label from predefined action categories (action classification [16,17]), to locate the starting and end positions of actions (action detection [18,19]), and to predict the future state of actions on the basis of incomplete action observations (action prediction [20,21]), the target of human action evaluation is to make computers automatically quantify how well people perform actions and further provide interpretable feedback for improving the motion of the human body. The field of human action evaluation research has recently begun to diversify because of its explosively emerging real-world applications, including healthcare and physical rehabilitation [22], skill training for expertise learners [23], as well as sports activity scoring [24], as shown in Figure 1.

(1) Healthcare and rehabilitation. Physical therapy is essential for the recovery training of stroke survivors and sports injury patients. As supervision by a professional provided by a hospital or medical agency is expensive, home training with a vision-based rehabilitation system is preferred since it allows people to practice movement training privately and economically. In computer-aided therapy or rehabilitation, human action evaluation can be applied to assist patients training at home, guide them to perform actions properly, and prevent them from further injuries.
(2) Skill training. Human action evaluation plays an important role in assessing the skill level of expert learners on self-learning platforms. For example, simulation-based surgical training platforms have been developed for surgical education. The technique of action quality assessment makes it possible to develop computational approaches that automatically evaluate the surgical students’ performance. Accordingly, meaningful feedback information can be provided to individuals and guide them to improve their skill levels. Another application field is the assembly line operations in industrial production. Human action evaluation can help to construct standardized action models related to different operation steps, as well as to evaluate the performance of trained workers. Automatically assessing the action quality of workers can be beneficial by improving working performance, promoting productive efficiency, and, more importantly, discovering dangerous actions before damage occurs.
(3) Sports activity scoring. In recent years, the video assistant referee (VAR) system has been introduced to some international sports competition events. The VAR serves as a match official who reviews the referee staffing’s decisions on the basis of video footage. Thus, decision making can be affected by contacting the referee on the field of play. Human action evaluation makes it possible to analyze the motion quality of athletes, judge the normalization of postures or body movements, and score the action performances automatically. The VAR system facilitates detailed assessments of sports activities and ensures the fairness of competition.

Human action evaluation relies on accurate human motion detection and tracking, action segmentation and representation, and efficient evaluation methods for measuring the quality of action performance. Almost all the challenges of human motion analysis also need to be tackled in action evaluation research, including the diversity and complexity of human body movement, action segmentation in long-duration video, and robust feature learning. Some factors increase the difficulties in this field of research, such as intra-class variations in scale, appearance, speed, and style, as well as several environmental factors, including poor illumination, camera motion, and cluttered backgrounds. 

In the last decade, quite a few reviews have been published to summarize the advancement of human action recognition. Conversely, a few action evaluation surveys have emerged, except for the work of [25], which is a recent thorough investigation on vision-based action understanding in application to assistive healthcare. The authors reviewed challenges and difficulties in advanced vision-based assistive healthcare and rehabilitation methods, available sensing devices for data acquisition, as well as some benchmark datasets. However, implementation methods and technical details in this field of research have not been addressed. This paper provides a comprehensive overview of recent human action evaluation studies. The review covers a variety of practical uses not only in the field of healthcare and physical rehabilitation, but also in application to skill training of expertise learners and sports activity scoring.

**Figure 2:**
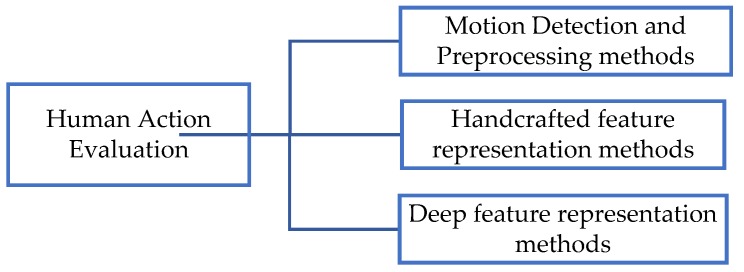
The framework of reviewed action evaluation research works.

Most of the reviewed studies have employed three processing stages in their implementation approaches. The first stage is to detect and segment human actions from long-duration video data. The second stage is to extract effective features for evaluating the quality of human actions in video segments, and the third stage is to develop an accurate assessment method for measuring the performance of human actions. The aggregation of segmental predictions to form a final decision on the complete activity is also performed in the final stage. Therefore, this study presents a thorough review of human action evaluation research from the perspective of its major processes and learning methodologies. Accordingly, the reviewed methods are divided into three parts: Motion detection and preprocessing methods, handcrafted feature representation methods, and deep neural network (DNN)-based feature methods for human action evaluation. The classification framework is shown in Figure 2.

Contrary to human action recognition, the research on action evaluation aims not only to recognize the action that is performed but also to provide a quality assessment result and feedback about how the action was performed. It is reasonable to assume that the quality of human action is directly dependent on the dynamic changes in human body movement, which can be represented by the motion trajectory and relative location relationship between joints or body parts, while it less influenced by environmental aspects, such as cluttered backgrounds or dynamic scenes. Thus, the skeleton data provide significant information for developing reliable quality assessment algorithms for action evaluation. Most related works have employed skeleton data that have been captured or detected from a depth or color camera for action evaluation research. This paper provides a comprehensive overview of recent methods of vision-based skeleton data analysis for action evaluation research, and we compare their performance on benchmark datasets are compared.

The contributions of this paper are fourfold:

(1) A clear problem definition is given of human action evaluation and clarify the differences among the three tasks of human activity understanding: Action recognition, action prediction, and action evaluation. To the best of the authors’ knowledge, it is the first review of action evaluation research that involves the diversity of real-world applications, such as healthcare, physical rehabilitation, skill training, and sports activity scoring.
(2) A thorough review of human action evaluation methods, including motion detection and preprocessing methods, handcrafted feature representation methods, and deep feature representation methods, are presented in this paper.
(3) Some benchmark datasets are introduced and compared with the existing research works on these datasets to provide useful information for those interested in this field of research.
(4) Moreover, some suggestions for future research are presented.

The remainder of this paper is organized as follows. In Section 2, the authors provide the problem definition of human action evaluation and clarify the difference among three tasks in research that aims to understand human activity, namely, action recognition, action prediction, and action evaluation. A thorough review of human action evaluation methods is presented in Section 3, including the skeleton data-based motion detection and preprocessing methods, handcrafted feature representation methods, and deep learning-based feature methods. In Section 4, the benchmark datasets and evaluation criteria for action evaluation are discussed. Finally, our conclusions are drawn in Section 5 and some suggestions are presented for future research.

## 2. Problem Definition

In human activity analysis, action is usually regarded as human body motion that involves the movements of joints, limbs, or body parts. In an early study on human activity analysis, Aggarwal [26] conceptually categorized human activities into four different levels: Gestures, actions, interactions, and group activities. Comparing the objectives of inference reveals that the research on human activity analysis can generally be divided into three tasks: Action recognition, action prediction, and action quality evaluation. These concepts are introduced below and clarify the differences among them in detail.

Human action recognition (HAR) [27,28,29,30] has been put forward to determine what the action is and when it is occurring. Two classic problems of action recognition have been extensively investigated in the past several decades: Action classification [31,32,33,34,35,36] and action detection [37,38,39,40,41]. The former aims to infer the action label for an input image or video, and the latter aims to detect the starting and ending times of all action occurrences from the video. Since particular types of actions can be recognized depending solely on limb morphology and the surrounding environment, still image or keyframe discovery-based human action recognition approaches have also been extensively researched in the last decade. 

Human action prediction (HAP) infers the action label of a video using a partially observed or incomplete action occurrence. The researchers in this field have commonly regarded it as a before-the-fact computer vision task [27,42,43]. It is important to predict abnormal behaviors early in public areas [44,45] or prevent dangerous driving behavior for the sake of traffic safety [46,47]. Human action prediction can be beneficial to some real-time monitoring applications, such as assistive living for elderly people, visual surveillance in public places, and autonomous vehicles. 

**Figure 3:**
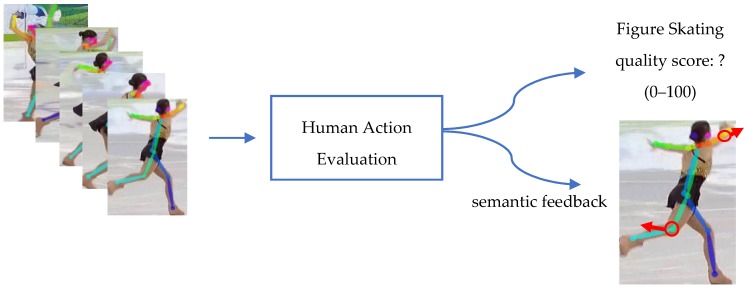
The task of human action evaluation (not only identifying action labels but also assessing the quality score and providing semantic feedback).

Human action evaluation (HAE) [1,2,3,4,5,6,7,8,9,10,48,49,50] aims to design computation models for representing the dynamic changing process of human movement and to develop evaluation techniques to measure the completion quality of human action. The target of action evaluation not only requires the recognition of the action performed but also more significantly, needs to provide a quality assessment of how the action was performed. Further, semantic feedback is presented understandably to facilitate the performers’ improvement of their deficiencies (see Figure 3).

The major differences between action recognition, action prediction, and action evaluation can be summarized in the following four points:

(1) From the perspective of the data to be processed, the temporal series of RGB video or depth sequences are a common data type in both action prediction and action evaluation. Still images are barely used for these two tasks. However, action recognition can usually be directly inferred from a still image or key frame of a video sequence. Furthermore, action prediction infers the class label with the condition that only parts of the action occurrence have been observed (the first part or middle part of the complete video). In contrast, accurate action evaluation estimation can only be achieved if complete human body movements have been observed and considered.
(2) Of the different targets among the three research tasks, human action prediction focuses on discovering abnormal events by forecasting unseen human actions and distinguishing anomalous actions from normal ones. Action recognition tries to classify a given still image or video sequence into a predefined action category or to find the start frame and the end frame of an action occurrence from a video sequence. Action evaluation has been presented to make a computer automatically give a good or bad assessment for action quality and provide interpretable feedback.
(3) Both human motion and environmental backgrounds provide important spatial–temporal information for action recognition and prediction. In contrast, the quality of action performance is directly determined by the dynamic changes in the human body or limbs, while it is less affected by environmental aspects. In particular, in the task of recognizing human–object interactions, such as people using telephones or playing instruments, the interacting objects provide an important clue for inferring the action category or intention. However, in a diving scoring application, the quality score of a diving performance involves the whole movement of the body and limbs of an athlete between taking off and entering the water. It matters whether the athletes’ feet are together and whether their toes are pointed straight throughout the whole diving process. The splash upon entering the water has a relatively minor impact only on the final stage assessment.
(4) For facilitating action recognition research, large-scale datasets have been collected and provided to researchers in this field, such as ImageNet, UCF-101, HMDB51, and Sports-1M. Efficient action recognition methods, especially recent deep feature approaches, including 3D convolutional networks, recurrent neural networks, and graph convolutional neural networks, have been trained from these large-scale annotated datasets. On the contrary, only a few small-scale datasets oriented to some specific applications, such as physical therapy, healthcare, sports activity scoring, and skill training, have been available for the research on human action evaluation. Small-scale annotated training samples, limited action categories, a stationary camera position, and the same backgrounds are common shortcomings among the published action evaluation datasets.

The following section presents a comprehensive review of the current human action evaluation research, including the detection and preprocessing methods of skeleton data (including detection, normalization, and alignment), handcrafted feature representation methods, and deep feature representation methods.

## 3. Overview of Human Action Evaluation Methods

Human action recognition has been investigated by researchers for a long time. Over the past few decades, computer vision-based action recognition techniques and methodologies have grown rapidly. An increasing number of reliable and efficient approaches have advanced quickly with the help of large-scale data and high-performance computation boosting. However, the research on action evaluation has achieved more interest recently because of its wide-ranging applications, such as assistive living for elderly people, medical therapy for patients, and skill training for expertise learners. Gordon [24] first put forward the problem of automated video assessment for human performance. The author proposed using computers that automatically analyze a performance recorded in video data. Ilg et al. [51] applied an earlier attempt to use the traditional feature extraction method to estimate the sports skill levels of individuals from 3D trajectory data obtained from a motion capture system. 

Human activity analysis in action evaluation research also faces challenges, such as the diversity and complexity of human movements, camera motion, occlusion, and background clutter. Similar key issues, including the motion detection and segmentation, feature extraction, and feature similarity measurement, have to be tackled for action evaluation. In early studies in this field, most research works [1,2,3,4,5,6,7] directly introduced and fine-tuned state-of-the-art action recognition approaches to deal with action evaluation problems. They either regarded action evaluation as a classification problem or simply replaced the optimization function of classification with regressions in the final stage without analyzing the essential differences between two tasks. Thus, the effect is far from meeting application requirements. Recently, deep neural network-based learning approaches, such as the 3D convolutional network (C3D) [52,53], long short-term memory network (LSTM) [54,55], and recurrent neural network (RNN) [56], have greatly boosted the action recognition performance on large-scale datasets. Some deep learning-based action evaluation studies have been attempted in the past few years. 

It is a natural and reasonable assumption that action quality directly depends on the dynamic changes in human body movements, as discussed in previous sections. Thus, skeleton data-based action quality assessment approaches have dominated in this field of research. In earlier studies, skeleton data could only be captured from a conditioned laboratory with environmental settings, such as a motion capture system (MoP), depth cameras, and stereo cameras. Thus, the evaluation performance was strongly restricted by the limitation of a small-scale training dataset. Now, with the help of start-of-the-art pose estimation techniques and methodologies, skeleton data can also be obtained from RGB images that facilitate the diversity of datasets. 

From the perspective of application domains, the motion representation and learning methodologies, the reviewed methods are summarized as presented in Table 1. Further, a detailed introduction about the implementation and technical details of typical methods are presented in the following subsections.

### 3.1. Detection and Preprocessing of Skeleton Data

#### 3.1.1. Skeleton Data Detection

Skeleton-based representations are suitable for modeling human actions by encoding the relationship between the joints or body parts, as well as the holistic body configuration. In order to develop an accurate action evaluation method, it is preferred that the dynamic changes in joints or body parts for analysis and action representation be used. The first important issue is the acquisition and preprocessing of skeleton data. In previous studies, RGB cameras were mostly used to create computer vision datasets. Skeleton data can be obtained from a particular motion capture system, for which performers have to wear markers near each joint to identify the motion by the positions or angles between the markers. As a result of the advent of depth-sensing cameras, such as the Microsoft Kinect sensor and Asus Xmotion, it is much easier to obtain skeleton data, thereby popularizing skeleton-based human representation. However, depth cameras severely suffer from occlusions, sensing distance, and poor performance in outdoor environments. 

With significant progress in recent pose estimation techniques and methodologies, skeleton data can now be estimated from RGB image data, which greatly facilitate the diversity of datasets. Traditional skeletonization models, such as the deformable part model [57] and flexible mixtures of parts model [58], have been replaced by deep neural network-based approaches since the advent of DeepPose. In 2014, Toshev and Szegedy [59] first applied deep neural networks for the precise localization of articulated joints for human pose estimation. They employed a two-stage process: A seven-layer generic convolutional DNN was constructed to regress the location of each body joint in the first stage, and in the second stage, DNN-based regressors were learned to refine the joint predictions by using higher-resolution subimages. DeepPose has achieved the best results, which have been superior to those of most traditional skeletonization models, and it has become the baseline for subsequent deep learning-based human pose estimation methods, such as OpenPose [60], AlphaPose [61], and DensePose [62].

**Figure 4:**
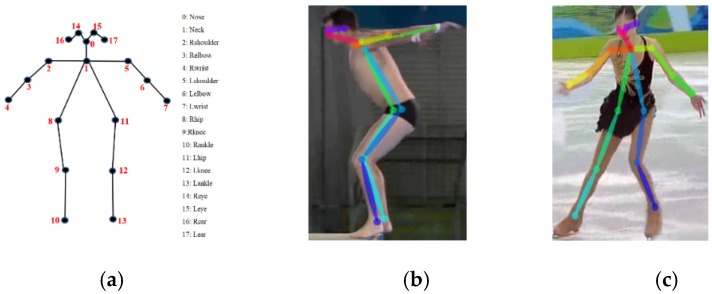
Skeleton model and detection examples of OpenPose [60]. (**a**) Eighteen-joint skeleton model of OpenPose; (**b**) example of diving; (**c**) example of figure skating.

OpenPose [60], the first real-time multi-person system, was developed by the perceptual computing lab of Carnegie Mellon University. It employs the architecture of two-branch multistage CNNs and uses part affinity fields (PAFs) to encode the location and orientation of limbs. These feature vectors sufficiently represent a global context and help to achieve high-quality greedy parsing results. OpenPose provides the functionality of 2D real-time multi-person keypoint detection (15- or 18- or 25-keypoint body/foot keypoint estimation), as well as the 3D real-time single-person keypoint detection. An example of an 18-keypoint skeleton model of OpenPose is shown in Figure 4a, including the nose, neck, right shoulder, right elbow, right wrist, left shoulder, left elbow, left wrist, right hip, right knee, right ankle, left hip, left knee, left ankle, right eye, left eye, right ear, and left ear. Some detection examples of diving and figure skating using the Olympic Sports Dataset [1] are illustrated in Figure 4b,c.

#### 3.1.2. Skeleton Data Preprocessing

The estimated skeleton data can often be noisy in cases of occlusion or cluttered backgrounds in realistic scenes. To obtain robust similarity quantization for fine-grained quality assessment, the preliminary treatment of skeleton data is noise filtering. Subsequently, normalization and alignment [63,64,65,66,67] are performed to deal with intra-class variations in the scale, transition, and rotation, as well as various appearances or motion styles.

In the case of occlusion or self-occlusion, the failure or false detection of the human body leads to the emergence of outliers of the joint’s coordinates in skeleton pose estimation that lead to an unreliable representation of human movements. Thus, the noise filtering process has been commonly performed on the original joint coordinates in most research works. The traditional image filtering techniques, such as Laplacian smoothing, Gaussian filtering, discrete cosine transformation (DCT), and discrete Fourier transformation (DFT), have been employed to transform the discrete coordinates of the joint trajectory. Consequently, zero values or sharply changing coordinates can be filtered out, and low-frequency components are preserved for the reliable detection of human body positions. 

Since both the height of persons and the photographing distance are quite different, the scale of the human body in different videos can be quite diverse. The original skeleton positions need to be normalized to a prototypical range for comparison. Without scaling, two motion instances with similar action quality may produce very different assessment results. The scale normalization process can be summarized in three steps. First, the middle of the left and right hip in the first skeleton of the sequence is chosen as a reference point. Then, the distance from the neck to that reference point is computed and defined as the normalized length. Finally, the position coordinates of joints are transformed by scaling with the normalized length. 

Another intra-class variation is that the spatial and temporal position of the human body can be very different located in various videos. The performer might not be in the same position relative to the camera, and the action video is usually untrimmed to include the preparing and ending duration of an action instance. Consequently, the skeleton positions need to be aligned in both the spatial and temporal dimensions. Since the position offset relative to the center of the camera will not be the same, the joints’ relative positions are computed by subtracting the coordinate of the hip center to obtain a skeleton-centric representation. After that, the rotation transformation is performed on the relative joints’ positions. The rotation angle θ is determined by the projection of the vector from the left hip to the right hip onto the x-axis. Then, each joint’s coordinate is transformed by rotating θ degrees to remove the impact of view variation. For temporal alignment, the traditional dynamic time warping (DTW) method has been used by some previous research works.

Most published skeleton-based action evaluation studies have performed the normalization and alignment process using the above-introduced methods. Paiement et al. [6] extracted skeleton data from Kinect’s depth data and preprocessed the skeleton data with scaling and spatial alignment. They employed diffusion maps, which constitute a graph-based technique with a quasi-isometric mapping of the original higher space to a reduced low-dimensional diffusion space, to reduce the dimensionality and deal with noise and outliers of skeleton data extracted from Kinect’s depth data. They validated their method by the assessment of gait on stairs. Pirsiavash et al. [1] employed the flexible mixtures of parts model and dynamic programming algorithm to find the best pose track in an Olympic diving or figure skating video. A discrete cosine transform was performed on the normalized positions of the joint trajectory, and the *k* lowest frequency components were used as high-level pose features for assessing the quality of human actions in videos. They found that low frequency feature extraction helped remove high-frequency noise due to pose estimation errors. In the work of [22], the authors conducted scaling and spatial alignment to normalize the positions of the joint trajectory, and relative positions and orientations were computed from the raw skeleton 3D time-series data. They also employed discrete cosine transformation to project joint positions and angles into the frequency domain. 

### 3.2. Handcrafted Feature Methods for Human Action Evaluation

The traditional action recognition’s two-staged processing inspired a similar framework that comprises action feature representation, and feature assessment learning was commonly used in the early action evaluation research. First, the local or holistic motion features are extracted to represent human actions through some handcraft-designed feature engines. Traditional feature detectors and descriptors, such as spatial–temporal interest points (STIP) [68], histogram of gradient (HOG) [69], histogram of optical flow (HOF) [70], scale-invariant feature transform (SIFT) [71], and motion boundary histogram (MBH) [72], have been commonly employed. Then, some research works formulated action quality assessment into an optimization framework in which action models are trained using the bag of words (BOW) [73] or the hidden Markov model (HMM) [74], and the corresponding evaluation functions are determined to assess the quality score of action features. The interpretable feedback is further provided to improve motion performance. From the perspective of major application fields, a detailed review of handcrafted feature methods in action evaluation research is presented in this subsection.

#### 3.2.1. Handcrafted Features for Physical Rehabilitation

Of the research on physical therapy or rehabilitation, Chen et al. [75] made an early attempt to develop a computational model for the quantitative quality evaluation of human body movement in a rehabilitation clinic setting. They modeled the evaluation function as a linear combination of 33 normalized kinematic attributes organized into seven categories—four activity level categories and three body-function-level categories—such as temporal profile, targeting, trajectory, velocity, and joint function. The RankSVM algorithm was modified and used to estimate the weights of different movements according to the clinician’s comparison of impaired subjects’ movement quality in pairs. The authors applied the learned kinematic impairment measure function to assess the reach and grasp of stroke survivors. The high Pearson correlations between the estimated results and the therapist’s decisions were achieved. 

Venkataraman et al. [76] proposed the use of the shape distribution of the reconstructed phase space called a dynamical attractor as the dynamical feature for modeling actions. They developed a shape-theoretic method to estimate the movement quality for single-marker home-based stroke rehabilitation. They mapped a one-dimensional time series to the reconstructed phase space and extracted the shape distribution to represent the dynamical shape feature of the mapped m-dimensional attractor. Then, the super vector regression function was trained to compute the quality score from dynamical shape features. The Pearson correlation coefficients achieved were better than those in the work on kinematic analysis in [75].

In the work of [77], Celiktutan et al. presented a graph-based physical exercise action evaluation method by modeling the spatiotemporal relationship between the skeleton joints by a chain graphical structure. They computed the inclination and azimuth angles of joints with respect to the torso basis, as well as the Euclidean distance between joints, to represent pose features. The pose sequence was modeled by a chain graphical structure and aligned by a graph matching technique. The matching energy was used to assess the performance of a given movement sequence aligned to the model sequence. 

In recent skeleton-based physical rehabilitation research, temporal alignment between skeleton sequences has been addressed for assessing the quality of physical exercise in several works. Antunes et al. [7] presented a visual and human-interpretable feedback system for guiding the user in the proper performance of certain actions and movements. First, skeleton data were preprocessed for the spatial and temporal alignment between a skeleton instance and the template counterpart. Then, by calculating the Euclidean distance of the 3D coordinates of body parts, the matching error between two skeleton sequences was quantified to assess the quality score. Finally, feedback proposals were automatically computed by minimizing the skeleton matching error. 

Baptista et al. [8] employed subsequence dynamic time warping to find the temporal alignment, and the temporal commonality discovery was presented to discover the interval of the subsequence with content that was similar to that of the template action. A qualitative evaluation was performed, and feedback proposals were provided in order to correct the user with respect to the template action. 

Paiement et al. [9] studied and compared the performances of different pose representations and HMMs with the dynamics of movement for the online quality assessment of human motion. They developed robust manifold representation and a first-order Markovian assumption to describe the dynamics of a human body pose. Their experimental results showed that the continuous-state HMM was better suited for describing motion dynamics than the classical discrete state HMMs when frame-by-frame deviation was measured for motion quality assessment. In their earlier work of [6], they introduced diffusion maps based on a nonlinear manifold technique to deal with noise and outliers for filtering skeleton data. To assess the quality of movements, they proposed two statistical models to represent the probability density function of the poses and dynamics of the skeleton, and they calculated the log-likelihoods of an observation compared with these models to represent the measures of pose quality and dynamics quality. They tested the proposed method on gait on stairs and demonstrated its generalizability to the movements of unknown subjects and ability to detect a range of abnormality types.

Elkholy et al. [78] proposed to compute handcrafted features from 3D skeletal data provided by depth sensors to detect and assess motion disorders in realistic scenarios. The proposed descriptor consisted of three medical justified features, including asymmetry, velocity magnitude, and Center of Mass (CoM) trajectory deformation, to encode both spatial and temporal characteristics of the motions. Then two probabilistic normalcy models of a Gaussian mixture model (GMM) and Kernel density estimation (KDE) were developed to model normal motion patterns, and the likelihood of a testing sequence was computed and used as a normalcy similarity measure. Furthermore, they trained a multiple linear regression with interactions model for predicting the assessment score to evaluate the degree of abnormality in performing an action. Experimental results demonstrated that the proposed methods can detect the abnormality in performing different activities for patients with different types of neuromusculoskeletal disorders and assess the quality of the action performance as well.

#### 3.2.2. Handcrafted Feature for Sports Activity Scoring

Some action evaluation studies have addressed automatic sports activity scoring based on the analysis of skeleton data. There are mainly two challenges to be tackled in this field of research. The first one is determining the means of extracting effective and robust features from pose sequences for action quality assessment, and the second one is establishing methods to measure the similarity of pose features to the tolerance of intra-class variations while distinguishing the inter-class confusions. Some research works have regarded action quality assessment as time sequences’ modeling and a similarity measurement problem, while others have approached it from the perspective of machine learning. 

Gordon [24] first presented the idea of automated assessment of human performance in video. Three key issues concerning the automatic video assessment problem have been addressed: The appropriate performance type, the necessary computer technology, and the way in which automation affects issues surrounding performance scoring. Then, they proposed the use of a motion tracking algorithm in computer vision to extract positions of the human body center from video and compute the difference in positions to assess several components of vaulting performance according to the Federation International de Gymnastique (FIG) Code of Points. Ilg et al. [51] applied an earlier work on constructing an action quality assessment dataset. They used VICON612—a motion capture system equipped with 11 cameras and 41 markers on the human body—to collect 14 karate kata videos performed by seven actors. Then, a hierarchical learning-based method to estimate skill levels from sequences of complex movements in sports was developed. In their learning method, three invariant key features, namely, angular velocity, curvature, and torsion of 3D trajectories, were computed to identify movement primitives first. A sliding window search was performed on a test video to find the subsequence that was the most similar to template features. Then, spatio-temporal morphable models (STMMs) were employed to learn generative models for different styles of movements. Finally, radical basis function (RBF) networks were trained with expert ratings to estimate the skill level of each sequence. 

Wnuk and Soatto [79] created the first diving dataset, FINA09, which was gathered from the finals of the diving event at the 13th FINA World Championships. The dataset consists of 68 diving videos performed by 12 different divers with 10 dive types, including 107B, 205B, and 307C. First, they applied background subtraction to obtain foreground regions and Kalman filtering to track the center of mass of the diver. Then, different pose descriptors, action modeling methods, and classification approaches were applied in an attempt to classify the dive types. The experimental results demonstrated that gradient orientation histogram-based pose representation was effective for dive type classification. The SIFT pose features computed after applying the foreground mask within a fixed square window size centered at the diver’s tracked location, divided into a 4 × 4 spatial bin configuration and 8-bin orientation, performed better than those without incorporating background subtraction, as well as HOG-HOF features. 

Pirsiavash et al. [1] collected 309 diving and figure skating competition videos to create a new MIT Olympic Scoring dataset. They proposed a general learning-based framework to assess the quality of human-based actions from videos. They extracted both low-level spatiotemporal filtering features that captured edges and velocities, as well as high-level pose features obtained from the discrete cosine transformation (DCT) of joint displacement vectors, and they estimated a regression model that predicted the scores of actions. Compared with the space–time interest points (STIPs) and DFT pose features, the DCT pose features achieved improved rank correlation between the estimated scores and the ground truths for both diving and figure skating.

Venkataraman [3] proposed computing multivariate approximate entropy to model dynamics in individual body joints and cross approximate entropy to model the interaction between body joints. They used the two-feature representations to quantify the dynamical regularity of human actions for action segmentation and diving action evaluation. In their experiment, the rank correlation was better than that in [1] for diving quality assessment. 

#### 3.2.3. Handcrafted Feature for Skill Training

In application to skill training, Sharma [80] applied an earlier attempt to design a fully automated surgical skill assessment framework from video data on the basis of Objective Structured Assessment of Technical Skills (OSATS). They used the STIP detector proposed by Laptev [68] and computed 162-dimensioned HOG-HOF descriptors to describe 3D video patches around each detected STIP. These descriptors were clustered by BOW. Then, the sequential motion texture features were computed by a gray-level co-occurrence matrix texture analysis to encode the sequential motion information. The experimental results demonstrated that the combination of local spatial–temporal features with sequential relations helped to improve the performance of automated OSATS assessments. 

Relevant studies were furthered in the works of Zia [81,82]. These studies also employed the STIP detector and HOG-HOF descriptor to extract local motion features. The K-means algorithm was used to cluster local features to obtain motion class time series. Then, three types of feature modeling, namely, symbolic features (HMM, BOW, and ABOW), texture features (motion texture and sequential motion texture), and frequency features (DFT and DCT), were applied in an attempt to represent motion features. In the final stage, the nearest-neighbor classifier was trained with the cosine distance metric for sequential feature selection to determine the skill level of surgical actions. They demonstrated that fine-grained analysis of motion dynamics in the frequency domain was more effective than other feature models in representing the skill-relevant information for surgical skill assessment. 

Recently, Fard et al. [83] presented a classification framework to automatically assess the expertise level of surgical learners. With the combination of a clinical viewpoint and motion characteristics, they proposed eight global movement features extracted from movement trajectory data, including (task completion time, path length, depth perception, speed, motion smoothness, curvature, turning angle, and tortuosity) extracted from movement trajectory data to quantify the movement pattern of surgical actions. These features were fed into three classification algorithms—k-nearest neighbor (k-NN), logistic regression, and support vector machine—to train a binary classifier that determined two skill levels: Expert and novice.

In summary, the criteria of quality assessment with a diversity of action categories are hard to unify, and some evaluation criteria are not quantifiable. It is difficult to design one mechanical feature engine to extract various kinds of patterns for all-class action evaluation. Furthermore, a handcrafted feature representation has been proved unsuitable for indicating the characteristics of complex activities in long-duration videos. These difficulties lead to large gaps between the estimated results and the ground-truth scores. The evaluation performance of handcrafted feature methods is far from satisfactory. 

### 3.3. Deep Feature Methods for Human Action Evaluation

With the popularity of using convolutional neural networks to tackle the problem of image representation and understanding in computer vision, many studies have taken an interest in developing deep learning methods for the analysis of human activity. Some typical learning networks, such as the 3D convolutional network (C3D) [84,85], long-short term memory (LSTM) [86,87], and the two-stream convolutional network [88,89], have been presented to extract deep features from human activities for action recognition. 

The C3D-based feature learning has extended convolutional feature extraction from the spatial dimension to both the spatial and temporal domain. It takes a video sequence as an input with three (x, y, t) dimensions, where x and y represent the position in the spatial domain, and t denotes the coordinates on the temporal axis. Then, three-dimensional convolution is performed in the learning network to extract the semantic spatio-temporal features to represent the dynamic patterns of human activity. Due to the large-scaled parameters and the high cost of storage capacity, researchers have further proposed decoupling a three-dimensional convolution into two separated branches—spatial and temporal—and named it a two-stream convolution network. One branch of the spatial network takes RGB images as an input and extracts the appearance information of human action, while another branch of the temporal network takes the input of optical images computed from consecutive frames to represent motion information. 

With regard to long-duration activity representation in video, there are two main types of approaches: The temporal aggregation methods and temporal relationship learning methods. In temporal aggregated representation, feature extraction is conducted on each frame or segment. Then, a pooling operation, such as max pooling or average pooling, is used to fuse all frame-level or segment-level feature vectors into final video-level features. In temporal relationship learning methods, a recurrent neural network, such as long-short term memory, is employed to model the temporal relationship among the frames or segments and to form the video-level representation.

#### 3.3.1. Deep Features for Physical Rehabilitation

In application to physical therapy or rehabilitation, Parmar [22] performed an earlier comparison of different learning techniques, including support vector machine, single- and double-layered neural networks, and boosted decision trees, to determine whether an action quality is good or bad. They trained a single-layered neural network with 9600 input neurons and 500 neurons in the hidden layer, as well as a two-hidden-layered neural network to classify actions. However, the accuracy of the neural network was inferior to that of the AdaBoost decision tree using the same skeleton feature representation, possibly because of an unreasonable configuration of networks and learning strategy. 

Recently, Vakanski et al. [90] created a physical rehabilitation movement dataset called UI-PRMD, which comprises 100 instances of 10 rehabilitation movements captured by a Vicon optical tracker and a Kinect camera. In their work of [91], they further proposed three end-to-end deep learning-based models, namely, CNNs, an RNN with two bidirectional layers of LSTM units, and hierarchical neural networks (HNNs) with five recurrent subnetworks, to encode the relationship between the joint coordinate data and quality assessment scores. Antunes et al. [92] introduced a labeled 3D skeletal dataset called AHA-3D for research on automatic fitness exercise assessment, and it comprises 79 skeleton videos of 21 subjects performing four different movements. They trained a two-stream CNN and an LSTM network separately as the baseline methods for the recognition and detection tasks. The high accuracy rates of 91% in recognition and 88.29% in detection were achieved. In addition, Blanchard et al. [93] presented a dataset consisting of 480 countermovement jumps and drop jumps to facilitate research on assessing anterior cruciate ligament (ACL) injury risk in female athletes from the perspective of computer vision.

#### 3.3.2. Deep Features for Sports Activity Scoring 

For sports activity scoring, Parmar also applied a prior attempt to introduce a deep learning method for assessing the action quality of athletes. In the work of [94], they utilized a C3D to extract spatio-temporal features to obtain clip-level feature vectors. Then, the average pooling and LSTM were employed to model the sequential relation between video clips and form the video-level feature representation. Finally, a super vector regression-based scoring estimation function was trained from the input of normalized video-level feature vectors. The experimental results showed that the average pooling performed well in assessing the quality of diving and vaulting videos in a comparison using the LSTM aggregated sequential relation representation. In a further investigation into whether actions have some commonalities among different categories, the AQA-7 dataset [95] was compiled for action quality assessment in sports videos. Using the same C3D-LSTM framework as that used in [94], the authors compared the performance of an all-action model with an action-specific model to test the hypothesis that knowledge transfer is possible in action evaluation. They demonstrated that with the LSTM aggregated video-level feature representation, the all-action model outperformed the single-action model. It is worth noting that C3D-SVR with the single-action model always achieved the best performance. However, the multi-action model outperformed any single-action model in the evaluation of unseen or novel action classes. Recently, in the work of [96], a multitask learning framework was introduced to tackle three action analysis tasks: Action recognition, commentary generation, and action quality assessment. They proved the effectiveness of C3D-AVG-MTL in capturing the inherent concept of action qualities, and they achieved a 90.44% correlation between the estimated results and the ground-truth scores. 

In their research on scoring figure skating activities in video, Xu et al. [97] contributed a figure skating Fis-V dataset that consists of 500 competition videos of 149 professional players and the average length of each video is approximately 170 seconds. They utilized the state-of-the-art deep architecture and proposed a multiscale convolution aggregation model with skip-LSTM for skating activity scoring. The two subnetworks of self-attentive LSTM and multiscale convolutional skip-LSTM were designed in this model: The former was used to learn the different weights of video clips and select important features for total element score regression, and the latter was employed to extract important local and global features while discarding redundant information for a total program component score regression. In [98], Xiang et al. proposed a segmental network called Pseudo-3D (P3D) to tackle the problem of sports activity scoring. They utilized the encoder-decoder temporal convolutional network (ED-TCN) [99] to divide diving videos into four segments: Jumping, dropping, entering the water, and water spray decaying. Then, three types of P3D ResNet networks [100], namely, serial, parallel, and composition of P3D, were used in an attempt to extract feature representation. The last layer of P3D was replaced by a fully connected layer with dropout for regression. They achieved significant improvement on the UNLV-Dive dataset and found that P3D CNN-based feature extraction on full video performed similarly when only water spray was involved. However, the segment-aggregated feature extraction could effectively model the criteria of scoring by weighting the contribution of different stages. 

Relevant studies have emerged quickly to diversify this field of research, including approaches such as ScoringNet [101,102] (a 3D CNN network for sports activity scoring) and SwingNet [103] (a hybrid deep neural network and recurrent neural network for assessing golf swing performance), as well as deep learning-based yoga asana recognition [104].

#### 3.3.3. Deep Features for Skill Training

In application to skill training, Wang et al. [105] first explored the development of deep learning methods for assessing surgical skill by analyzing multivariate time-series data of motion kinematics. They proposed a 10-layered convolutional neural network that comprises five types of operations, namely, convolution, pooling, flattening, full connection, and softmax regression, to extract the intrinsic motion dynamics for automatic skill assessment. The network was trained with the loss of the difference between the estimated results and the truth labels by minimizing the multinomial cross-entropy cost. Fawaz et al. [106] designed a CNN method to evaluate surgical skills from kinematic data. In this learning architecture, 76-dimensional kinematic data were captured and inputted into the network. The first layer captured gesture information, and the second layer acquired global information related to surgical skill level. A global average pooling operation was employed after convolution to identify the regions that contributed the most to skill determination, and a fully connected softmax layer predicted the class label (N, I, and E, which denote novice, intermediate, and expert, respectively). 

Without the provision of kinematic data or tool motion data from a robotic surgical system, Funke et al. [107] first proposed a video-based surgical skill assessment method using a deep neural network. They fine-tuned a pretrained 3D ConvNet to extract spatio-temporal features from video snippets and used a temporal segment network to resolve ambiguities in single video snippets for the task of surgical skill assessment. Doughty et al. [108] presented a pairwise deep ranking method for skill determination in video and applied the proposed method to a variety of tasks, such as knot tying, needle passing, and suturing in surgery; dough-rolling; drawing; and chopstick-using. Two-stream CNN was employed to extract the spatio-temporal feature representations for all pairs of videos, and pairwise ranking and similarity were computed in the loss function. In their further study of [109], a new Bristol everyday skill tasks (BEST) dataset was introduced for researching skill determination in long videos. They proposed a rank-aware temporal attention network that comprised I3D feature extraction, attention-filtering representation, and a ranking process, among which multiple attention filters were employed in the attention module to discover important parts in the long video for skill determination. The network was trained with the three types of loss: ranking loss, disparity loss, and rank-aware loss; it achieved state-of-the-art performance on the Epic-Skill and BEST datasets. 

A novel RNN-based spatial attention model was proposed in [110] to capture temporal transition patterns of attention combined with high-level task-related features for better skill assessment in video. The learning framework consisted of two RNNs, one for estimating the spatial attention for each frame and the other for skill score estimation. Three processing modules were designed to realize estimation, among which the feature encoding module extracted deep appearance–motion features from RGB and optical flow images. The attention pooling module combined appearance–motion features with task-related features, as well as the temporal relationship between attention. The temporal aggregation module modeled the temporal transition of actions to form the final accumulated representation. The experiments on an infant grasp dataset and BEST dataset [109] demonstrated that considering attention can be helpful for the automatic skill assessment of chopstick-using, dough-rolling, drawing, and surgery. 

Although significant progress has been achieved for human action evaluation by deep learning-based feature representation, some key issues deserve to be further studied. On the one hand, the performance on practical application tasks, such as sports activity scoring, physical exercise training, and skill level determination, is below current application requirements. This deficiency is possibly because most published deep learning methods have employed mechanical equal-sized division of long-duration video for the sake of reducing the parameter scale of the learning network. The important temporal information might be lost because of over-segmentation or false segmentation of actions. On the other hand, most of the above-mentioned research works have simply duplicated existing deep feature models designed for action recognition and directly implanted them into the task of action evaluation. Thus, it is difficult to develop a reliable action evaluation method without distinguishing the intrinsic difference between the two problems.

## 4. Benchmark Datasets and Evaluation Criteria

### 4.1. Datasets

Large-scale datasets with diverse actions or subjects for validating action evaluation algorithms are still lacking. Most public datasets are oriented toward particular applications, and a limited number of training samples have been collected. As the construction of an action evaluation dataset requires domain experts to provide ground-truth annotations for data, it is difficult to build a universal dataset comprising multiple action categories and massive training data. As an alternative, researchers have created their datasets manually for some specific tasks.

#### 4.1.1. Physical Rehabilitation Datasets

Most physical rehabilitation-relevant datasets remain publicly unavailable because of two major factors. One is the privacy issue concerning patients’ or elderly people’s private activities, and the other is about the property rights reserved by some medical organizations or funding companies. The five publicly available datasets are introduced dedicated to this field of research: University of Bristol’s AI SPHERE-Staircase2014 [111], SPHERE-Walking2015 [112], SPHERE-SitStand2015 [113], the University of Idaho-Physical Rehabilitation Movement Data (UI-PRMD) dataset [114], and the AHA-3D dataset [115]. These publicly available datasets are summarized by the numbers of action categories, persons, and samples, as well as the action classes and the provision of data modality, as shown in Table 2. 

Paiement et al. [6,9] have devoted themselves to the motion quality assessment of physical therapy and rehabilitation. They presented the SPHERE-Staircase2014, SPHERE-Walking2015, and SPHERE-SitStand2015 datasets successively for evaluating action quality assessment methods that can be used to support the physical activity of stroke or sports injury patients at home. The SPHERE-Staircase2014 dataset [6] includes 48 sequences of walking upstairs performed by 12 individuals captured by an Asus Xmotion RGB-D camera placed at the top of the stairs in a frontal and downward-looking position. The SPHERE-Walking2015 dataset [9] includes 40 sequences of 10 individuals walking on a flat surface. This dataset was captured by an Asus Xmotion RGB-D camera placed in front of the subject. It contains normal gaits and two types of abnormal gaits to simulate stroke and Parkinson disease patients walking under the guidance of a physiotherapist. The SPHERE-SitStand2015 dataset [9] includes 109 sequences of 10 individuals performing two actions—sitting down and standing up—in a home environment. Instead of the Asus Xmotion RGB-D camera, a Kinect V2 camera was used to track the skeleton for movements. Two types of abnormal movements and normal motions are included.

Vakanski et al. [90] presented the UI-PRMD dataset of human body movements for physical rehabilitation exercise. They used a Vicon optical tracker and a Microsoft Kinect sensor to capture the positions and angles of full-body joints. For data collection, 10 subjects were recruited to perform 10 different movements repetitively, including the deep squat, hurdle step, inline lunge, side lunge, sit to stand, standing active straight leg raise, standing shoulder abduction, standing shoulder extension, standing shoulder internal–external rotation, and standing shoulder scaption. 

Recently, the AHA-3D dataset [92] was collected for assessing the fitness level of seniors during exercise. This dataset provides 3D skeletal data acquired from Kinect v2 and color images from RGB cameras. A sample of 21 subjects consisting of 11 young individuals and 10 elderly individuals (5 males and 16 females) performed four different motions: 30-second chair stand, 8-feet up and go, 2-minute step test, and unipedal stance. The result was the capture of 79 skeleton videos.

#### 4.1.2. Sports Activity Scoring Datasets 

In the research on automatic sports activity scoring, several sports activity datasets have been collected from international competition events on diving, figure skating, vaulting, and others. Several publicly available benchmark datasets are introduced, including the FINA09 diving dataset [79], MIT Olympic scoring dataset [1], UNLV AQA-7 dataset [95], MTL-AQA dataset [96], and Fis-V dataset [97], etc. The details of these benchmarks are presented in Table 3 from the perspective of action categories, the number of samples, data source, viewpoint, and variation in the background.

The FINA09 diving dataset [79] was collected from the 13th FINA World Championships of diving, and it contains 68 annotated diving videos in total, with 12 athletes each performing about six unique dives. Each dive was recorded from front and side viewpoints. The ground-truth score of each dive was obtained by the product of the execution score multiplied by the difficulty score.

The MIT Olympic Scoring dataset [1] is made of 159 diving videos and 150 figure skating videos captured from the Olympic competition and World Championship events on YouTube. The frame rate of diving is 60 frames per second (fps), about 150 frames per video, and the AQA score ranges from 20 (worst) to 100 (best). The frame rate of the figure skating video is 24 fps, about 4200 frames per video, and the AQA score ranges from 0 (worst) to 100 (best). The front and side viewpoints provided for diving, while severe changes of view exist in figure skating. 

The UNLV AQA-7 dataset [95] comprises 1189 videos of seven action categories: 370 singles diving-10m platform, 88 synchronous diving-3m springboard, 91 synchronous diving-10m platform, 176 gymnastic vaulting, 175 skiing, 206 snowboarding, and 83 trampoline videos were captured from Summer and Winter Olympics events on YouTube. There is a difference in the average length of videos among the seven action categories, ranging from 87 (vaulting) to 634 (trampoline). Severe changes in view exist in vaulting, skiing, and snowboarding, while small and negligible variations in viewpoint exist in trampoline and diving videos. The ground-truth score of each action is obtained by the product of the execution score multiplied by the difficulty score.

The MTL-AQA dataset [96], the largest diving dataset to date, was collected from 16 international competition events and contains 1412 samples of single or synchronized diving performances from 10 m platform and 3 m springboard diving. The variations in this challenging dataset include viewpoint, male and female divers, and cluttered background. Each sample is annotated with three labels: the action quality assessment score, action category, and commentary.

Xu et al. [97] proposed the Fis-V dataset, which comprises 500 figure skating videos collected from high-standard international skating competitions, including the NHK Trophy (NHK), Trophee Eric Bompard (TEB), the Cup of China (COC), and the Four Continents Figure Skating Championships (4CC). The frame rate is 25 fps, about 4300 frames per video, performed by 149 athletes from 20 different countries. Each video is annotated by two scores: Total element score (TES) and total program component score (PCS). 

To facilitate golf swing analysis, McNally et al. [103] introduced GolfDB, a benchmark video database for the novel task of golf swing sequencing. It was collected from 580 YouTube videos and consists of professional golfers from the PGA, LPGA, and Champions Tours, totaling 248 individuals with diverse golf swings. A total of 1400 high-quality golf swing videos and over 390k frames captured from multiple camera angles and multiple backgrounds were extracted. 

Since yoga exercises provide physical, mental, and spiritual benefits to people, yoga learning and self-instruction systems are becoming popular across the globe. Some researchers have investigated computer-assisted self-training systems for improving the performance of yoga exercises. Recently, Yadav et al. developed a deep learning algorithm to recognize various yoga asanas. To this end, a yoga dataset called YogaVidCollected [120] was created using an HD 1080p RGB webcam that recorded 15 individuals performing six yoga asanas (i.e., Bhujangasana, Padmasana, Shavasana, Tadasana, Trikonasana, and Vrikshasana). In total, 88 videos with approximately 111,750 frames were collected in an indoor environment, and most were performed at a 4–5-meter distance from the front of the camera.

#### 4.1.3. Skill Training-Related Datasets

In research on skill training, some datasets have been created for evaluating the action performance of expertise learners for particular tasks, such as surgical skill training and monitoring and assisting the daily lives of elderly people. Several publicly available datasets have been introduced in this application area, including the JIGSAWS dataset [121,122,123], EPIC-Skills 2018 dataset [124], BEST 2019 dataset [125], breakfast action dataset [126], and the ADL dataset [127], as illustrated in Table 4.

The JIGSAWS dataset [122,123] was captured by a da Vinci Surgical System and consists of kinematics and video data from eight participants for three robotic surgical tasks: Suturing (SU), knot tying (KT), and needle passing (NP). All subjects repeated each surgical task five times. As some trials (1 for SU, 4 for KT, and 12 for NP) were unusable as a result of corrupted data recordings, the final dataset consists of 39 samples of SU, 36 samples of KT, and 28 samples of NP. This dataset was collected for the Language of Surgery project at the John Hopkins University site. The project is dedicated to developing models for analyzing surgical motion data and applying these models to teaching and assessing action performance of surgical trainees.

The EPIC-Skills 2018 dataset [108,124] comprises 216 samples of six tasks from two published and two newly recorded datasets: 103 samples of knot tying, needle passing, and suturing from JIGSAWS are used for surgical skill assessment, and 33 dough-rolling samples performed by 33 persons from CMU-MMAC dataset are used for assessing the pizza making activity. Another two tasks, drawing and chopstick-using, were captured by a high-resolution camera with a single position and background.

Since the limitations of the EPIC-Skills dataset of a single view and scarcely cluttered background result in scenes that are far from realistic of applications, a novel skill assessment dataset—Bristol everyday skill tasks (BEST) 2019 [109,125]—was collected. The BEST dataset comprises 500 videos of five skill tasks: Scrambling eggs, braiding hair, tying a tie, making an origami crane, and applying eyeliner. The videos were retrieved and compiled from YouTube. Therefore, the recording view and background may differ. Each video is annotated with a class label of B, I, or E, which indicate beginner, intermediate, and expert, respectively. Then, pairwise annotation is performed on 40% of the possible pairs for skill ranking. 

Another practical application of a skill training system is to provide living assistance for elderly people, protecting them from dangers or accidents in their daily life. Several datasets involving daily activities, such as breakfast preparation and cooking, have been created, for example, the Breakfast Actions dataset [126], ADL dataset [127], MPII cooking dataset [128], Charades [129], and EPIC-KITCHEN [130]. The breakfast actions, MPII cooking, and epic-kitchen datasets are recorded cooking activities. The ADL and Charades datasets contain hundreds of action classes and thousands of video sequences involving human daily lives in order to facilitate a computer vision system-based analysis of human daily activities. 

### 4.2. Performance Evaluation Criteria

In the published research, non-uniform evaluation criteria have been adopted for validating action evaluation algorithms since different problem definitions have been formulated in different application fields. In physical rehabilitation, two-class classification accuracy, namely, normal and abnormal movements, is commonly used as the evaluation criteria. A regression problem is generally formulated in sports activity scoring. Thus, the precision of the regression that measures the similarity between the prediction scores and ground-truth scores is determined to verify action quality assessment algorithms. Nevertheless, the ranking accuracy, classification, and regression are all used for validating the effectiveness of skill assessment methods. 

As physical rehabilitation entails private activities and property rights restrictions, a few datasets in this field are publicly available. The published research works have employed diverse evaluation methodologies and verified the proposed algorithms on their own datasets. Therefore, it is difficult to compare them using a unified criterion. Paiement et al. [6,9] formulated an online quality assessment for abnormal events detection. They developed a statistical normal movement model and measured how much a movement deviates from normal. They achieved 84% of abnormal events detection of gait on stairs on the SPHERE-Staircase2014 dataset, and the best AUCs were 1.00, 0.99, and 1.00 for walking, sitting, and standing on the SPHERE-Walking2015 and SPHERE-SitStand2015 datasets with the selected model parameters. Elkholy et al. [78] developed two probabilistic normalcy models of a Gaussian mixture model (GMM) and Kernel density estimation (KDE) to assess physical movements, and the best AUCs achieved were 1.00 for walking, sitting, and standing on the SPHERE datasets using their GMM model. With their KDE model, the best AUCs were 1.00 for walking, sitting, and standing and 0.98 for gait on stairs. They also employed an equal error rate, detection rate at a 1% false acceptance rate, and detection rate at a 5% false acceptance rate to evaluate the performance of the proposed method. Liao and Vakanski [90,91] introduced the UI-PRMD dataset and developed a deep learning framework for assessing physical rehabilitation exercises. They used and compared the regression performance of three deep learning networks—CNN, RNN, and HNN—as well as four metrics: Euclidean distance, Mahalanobis distance, DTW distance, and GMM log-likelihood.

In sports activity scoring, the performance of an action quality assessment method is commonly evaluated by the similarity between the predicted results and the ground-truth scores. The Spearman rank correlation coefficient or Pearson’s correlation coefficient is commonly employed to measure the similarity: The higher the value, the better the performance. The performance of some state-of-the-art methods on the MIT Olympic Scoring dataset and UNLV AQA-7 dataset are presented in Table 5. A comparison of performances on the MIT dataset makes it apparent that deep learning methods [94,95,97,98,101,102] significantly improve the performance of sports scoring estimation compared with handcrafted approaches [1,3]. The best performance on MIT diving improved to 0.86, and the figure skating was 0.59. With a larger dataset, UNLV AQA-7, which has more than one thousand videos, the best rank correlations are 0.84 and 0.7 for the diving and vaulting videos, respectively.

The skill assessment approaches commonly employ three types of evaluating criteria: Classification accuracy, score estimation accuracy, and rank accuracy. For example, three expertise levels, namely, expert, intermediate, and novice, are defined in the JIGSAWS dataset, and the classification rate of accurate predictions the class label (E, I, N) is regarded as the classification accuracy. Most published research works [83,105,116,117,118] report their classification accuracy as detailed in Table 6. A small selection of works, except for [118], have reported the correlation coefficient between the prediction scores and ground-truths as score estimation accuracy. Some researchers have suggested that the ranking of videos is more suitable than estimating an objective score [108,110]. Therefore, rank accuracy, which is defined as the percentage of correctly ordered videos in a ranking, has been employed to evaluate skill determination algorithms.

## 5. Conclusions and Discussion

In this paper, a comprehensive review was carried out of recent computer vision-based approaches for human action evaluation research. The relevant literature was summarized according to several key issues of action evaluation research, including motion representation based on skeleton data detection and preprocessing, handcrafted feature methods, and deep feature methods in different real-world applications. The benchmark datasets collected from the field of physical rehabilitation, sports activity scoring, and skill training were described. Useful information was provided to facilitate the researchers’ selection of suitable datasets for their studies. Furthermore, the effects of published action evaluation research were discussed by comparing their feature learning and assessing methods, as well as their performance on several benchmark datasets. The authors conclude the study on human action evaluation research with the following observations:

(1) Most existing research works have directly employed traditional machine learning or state-of-the-art deep learning methods in the field of action recognition to tackle the problem of action evaluation. Thus, it is difficult to develop reliable action quality assessment algorithms without distinguishing the intrinsic difference between these two problems.
(2) In the application of sports activity scoring, deep feature representation methods have been proved superior than handcrafted feature methods in their performance on benchmark datasets. Specifically, most of deep-learning methods significantly improve the scoring estimation results on MIT Olympic Scoring dataset than handcrafted approaches. The best correlation coefficient of dive scoring has been improved to 0.86, and the figure skating is 0.59 as introduced in Table 5. The reason is probably because that it is difficult to design one mechanical feature engine to extract various kinds of patterns for multiple-class action evaluation. Therefore, handcrafted feature representation is unsuitable for indicating characteristics of complex activities in long-duration video. On the other hand, most of deep learning methods have employed mechanical equal-sized division on long-duration video for the sake of reducing the parameter scale of learning networks. The important temporal information might be lost as a result of over-segmentation or false segmentation.
(3) In skill training, both the handcrafted and deep learning feature methods achieved high classification accuracy on the JIGSAWS dataset as presented in Table 6. However, a three-category of (E, I, N) classification evaluation is rather simple to evaluate the different methods. More appropriate evaluation criteria, such as rank accuracy and score prediction, deserves further investigation. In physical rehabilitation, a few datasets have been publicly available due to private activities and property rights. The reviewed studies employed diverse evaluation criteria, such as an abnormal events detection rate, equal error rate, and a detection rate with a false acceptance rate, on their own datasets. It is difficult to compare them in a unified criterion. The pro and cons of different methods remains to be further observation.
(4) The deep feature representation methods have significantly improved the performance on several benchmark datasets. However, their accuracy and efficiency are far from satisfactory and below the current application requirements.
(5) There is still a lack of large-scale annotated datasets with a diversity of action categories and application fields. This is mainly because of the great labor cost of the domain experts’ professional annotation. There is also a lack of unified evaluation criteria to validate the effectiveness of the proposed methods.

Although the difficulties and challenges remain in action evaluation research, such as intra-class variation in diversity and complexity of human movement, camera motion, view changes, occlusion, and background clutter, the performance of deep learning-based feature representation has improved. There are still some key issues that must be addressed for significant progress.

(1) It is reasonably assumed that the quality of human actions is directly determined by the dynamic change in human body movement rather than environmental factors. Thus, accurate skeleton data detection and deep feature representation methods based on skeleton data are the key issues in the development of reliable quality assessment algorithms for human action evaluation.
(2) The segmentation of long-duration video on the basis of primitive action semantics and the representation of temporal relationships between action segments are important topics of future deep architecture research for human action evaluation. Most previously published deep learning methods have employed an equal-sized division of video to reduce the parameter scale of learning networks. The important temporal information might be lost as a result of over-segmentation or false segmentation.
(3) The semantic granularity of the evaluation models needs to be further studied. Most existing studies have adopted a unified regression function to assess all action categories. Thus, the evaluation accuracy has significantly decreased under circumstances of unequally distributed training samples. Furthermore, an all-action regression model is not capable of assessing the quality of unseen actions. Whether a specific-action model or an all-action model is more suitable for evaluating the quality of actions and whether knowledge transfer can be adapted to train a unified evaluating model across action categories are promising directions that deserve study.

## Figures and Tables

**Table 1:** The reviewed research works of human action evaluation.

Applications Methods	Physical Therapy	Sports Activity Scoring	Skill Training
Skeleton or kinematic data-based methods	[6,7,8,9,22,90,91,92]	[1,2,3,4,5,48,51,104]	[83,105,106,116,117,118]
Handcrafted feature learning methods	[6,7,8,9,75,76,77,78]	[1,2,3,4,5,24,48,49,51,79]	[23,50,80,81,82,83,116,117,118]
Deep feature learning methods	[22,90,91,92,93]	[94,95,96,97,98,101,102,103,104]	[105,106,107,108,109,110]

**Table 2:** Physical rehabilitation dataset.

Dataset Name	#Action Categories	#Persons	#Samples	Data Modality
SPHERE-Staircase2014 dataset [111]	1	12	48	Depth sequences, skeletons
SPHERE-Walking2015 dataset [112]	1	10	40	Depth sequences, skeletons
SPHERE-SitStand 2015 dataset [113]	2	10	109	Depth sequences, skeletons
UI-PRMD dataset [114]	10	10	100	Positions and angles of body joints in the skeletal models
AHA-3D dataset [115]	4	21	79	3D skeletal sequences, RGB images

**Table 3:** Sports activity scoring dataset.

Dataset Name	#Action Categories	#Samples	View of Samples	Background of Samples
FINA09 diving dataset [79]	1	68	Front, side	Same
MIT Olympic Scoring dataset [1]	2	309	Variations	Same
UNLV AQA-7 dataset [95]	7	1189	Severe changes	Different
MTL-AQA dataset [96]	1	1412	Severe changes	Different
Fis-V dataset [97]	1	500	Severe changes	Different
GolfDB dataset [119]	1	1400	Multiple views	Different
YogaVidCollected dataset [120]	6	88	Small changes	Same

**Table 4:** Skill training-related dataset.

Dataset Name	#Action Categories	#Samples	Data Modality	View of Samples	Background of Samples
JIGSAWS dataset [122]	3	103	Kinematics data, video data	two left and right cameras	Same
EPIC-Skills 2018 dataset [124]	6	216	Video data	single view	Different
BEST 2019 dataset [125]	5	500	Video data	Severe changes	Different
Breakfast Actions database [126]	10	1989	Video data	3–5 cameras	Different
ADL dataset [127]	18	440	Video data	170-degree first-person view angle	Different

**Table 5:** Action evaluation performance of methods on sports scoring dataset. The superscript D indicates a deep learning method.

Methods	Year	MIT Olympic Scoring Diving	MIT Olympic Scoring Skating	UNLV AQA-7 Diving	UNLV AQA-7 Vault
[1]	2014	0.41	0.35		
[3]	2015	0.45			
[94] ^D^	2017	0.74	0.53	0.79	0.68
[95] ^D^	2018			0.61	0.67
[101] ^D^	2018		0.57	0.80	0.70
[102] ^D^	2018	0.78		0.84	0.70
[98] ^D^	2018	0.86			
[97] ^D^	2019		0.59		

**Table 6:** Action evaluation performance of methods on the JIGSAWS dataset. The superscript D indicates a deep learning method.

Method	Year	Evaluation Criteria	Action Categories		
			Suturing	Knot Tying	Needle Passing
[116]	2012	Classification accuracy	97.4%	96.2%	94.4%
[117]	2017	Classification accuracy	89.7%	96.3%	61.1%
[118]	2018	Classification accuracy	100%	99.9%	100%
		Score prediction (Correlation Coefficient)	0.75	0.63	0.46
[83]	2018	Classification accuracy	89.9%	95.8%	82.3%
[105] ^D^	2018	Classification accuracy	93.4%	89.8%	84.9%
[107] ^D^	2019	Classification accuracy	100%	-	96.4%
[108] ^D^	2018	Rank accuracy	70.2%		
[110] ^D^	2019	Rank accuracy	73.1%		
